# Synthetic immunotherapy induces HIV virus specific Th1 cytotoxic response and death of an HIV-1 infected human cell line through classic complement activation

**DOI:** 10.1186/1743-422X-10-107

**Published:** 2013-04-04

**Authors:** Olga Pleguezuelos, Gregory A Stoloff, Wilson Caparrós-Wanderley

**Affiliations:** 1 Research and Development, SEEK, 45 Beech Street, London, EC2Y 8AD, UK

**Keywords:** HIV, Immunotherapy, CD8+, Antibodies, Complement, Polypeptides, Vaccine

## Abstract

**Background:**

This manuscript describes the development of a novel synthetic immunotherapy (HIV-v) composed of four multi-epitope polypeptides targeting conserved regions in the Nef, Rev, Vif and Vpr viral proteins. Immunogenicity and cytotoxicity of HIV-v are discussed.

**Methods:**

Short conserved T-cell multi-epitope regions were identified *in silico* in the HIV proteome. The immunogenicity of the identified HIV-v polypeptides was assessed *in vivo* by immunisation of C57BLK6 mice transgenic for HLA-A*0201. Splenocytes from immunised animals were exposed *in vitro* to soluble HIV-v polypeptides or to syngeneic (T1) or allogeneic (Jurkat) cells transfected with these polypeptides. Specific T-cell reactivity was assessed by cell-based IFN-γ ELISA. Virus specific CD3 + CD8+ IFN-γ+ recall responses were also determined by flow cytometry following *in vitro* exposure of splenocytes from immunised mice to syngeneic (T1) and allogeneic (H9) cells infected with HIV-1 strain IIIB. HIV-v specific antibodies were quantified by ELISA whilst antibody mediated anti-viral immunotherapeutic effect on T1 cells infected with a laboratory adapted and a primary isolate of the HIV-1 virus was assessed in a LDH-based complement mediated lysis assay.

**Results:**

HIV-v elicited antigen-specific IgG and IFN−γ responses against the synthetic polypeptides in the formulation. HIV-v specific T cells recognised polypeptides presented either as soluble antigen or complexed to HLA-A*0201 following natural processing and presentation by syngeneic human T1 cells. Moreover, the CD3 + CD8+ component of the response recognised syngeneic T1 cells naturally infected with HIV-1 in a virus-specific and MHC restricted-manner. The HIV-v specific IgG response was also able to recognise human T1 cells naturally infected with HIV-1 and induce cell death through classic activation of complement.

**Conclusions:**

HIV-v induces a vaccine-specific type I immune response characterised by activation of effector CD8+ T cell and antibody responses that recognise and kill human cell lines naturally infected with a laboratory adapted and a primary isolate of the HIV-1 virus. The data supports the hypothesis that alternative HIV protein targets can be effectively used to prime both cellular and antibody immune responses of clinical value in the prevention and treatment of HIV infection.

## Background

Human Immunodeficiency virus (HIV) is the causative agent of AIDS. Worldwide, 25 million patients have died so far and over 34 million people are currently infected. Combined antiretroviral therapy has transformed the treatment of HIV/AIDS and extended patients’ life-expectancy. However, the long term-nature of this treatment is associated with severe toxic side-effects, limited compliance, development of resistance and high cost [1-3]. The only hope to control the continuous spread of HIV is to develop a vaccine or therapy that it is not only effective but affordable. In 2011 only 54% out of the 14.8 million people eligible for antiretroviral therapy were receiving it. The UNAIDS estimates that $6.8 billion a year will be required by 2015 to ensure access to treatment, care and support for 15 million people living with HIV/AIDS [4].

Despite the urgent need and the global scientific efforts, a vaccine to prevent HIV infection remains elusive due to the high diversity of the virus, its ability to evade the immune response and the lack of animal models in which to test the efficacy of the vaccine [5]. Attempts at developing prophylactic and therapeutic vaccines have been made using life attenuated or inactivated virus, or delivering HIV antigens as DNA vaccines or within viral vectors to induce a cytotoxic T cell response and/or a neutralising antibody response [5,6]. Preventative and therapeutic peptide vaccines against HIV are a safe and low cost alternative to anti-retroviral drugs and conventional vaccines. The focus has been to identify short conserved regions within the viral protein sequences, from as little as five conserved aminoacids in the highly variable Env gp160 protein [7] to single longer multiepitope peptides within Oyi, a Tat variant found in HIV African patients that did not progress to AIDS [8]. Others have selected multiepitope peptides derived from several viral proteins (Env, Gag and Nef) [9]. However, the reality remains that despite encouraging results in animal models [8,10,11], the immunogenicity of peptide vaccines in humans trials remains modest [9,12] highlighting the lack of correlation between the animal models and human trials.

Over the last 20 years most HIV candidate vaccines have exploited the immune responses naturally developed against specific viral antigens during HIV infection. These included antibody responses against Env [13-16] or more recently, cellular immune responses to Tat and Nef [17,18]. The findings of the RV144 trial, in which a combined regimen of T and B cell vaccines was modestly effective in preventing HIV infection [19], suggest that targeting both antibody and cellular immunity against HIV might constitute a better approach to induce protection, particularly if, as suggested elsewhere [20], this immunity is targeted to conserved regions within the HIV proteome.

In this report, we describe a novel candidate immunotherapy (HIV-v), containing four synthetic polypeptides derived from conserved immunoreactive regions of three accessory proteins Vif, Vpr, Nef and the regulatory protein Rev. The vaccine was prepared in Montanide ISA-51, a water-in-oil adjuvant that potentiates the immunogenicity of the peptide preparation. We provide evidence that HIV-v induces specific CD8+ T cell and IgG responses capable of recognising and killing a human cell line infected with a laboratory adapted and a primary isolate of the HIV-1 virus.

## Results and discussion

### Polypeptide selection

A major hurdle in HIV immunotherapy development is viral sequence variability [21]. The aim of the analysis was to identify highly conserved domains, containing a high number of *in silico* predicted T cell epitopes, within HIV proteins. It was not our aim to identify highly conserved HIV proteins towards which high frequency natural immune responses are directed during infection. Protein sequences from HIV-1 and HIV-2 strains were included in the analysis since our aim was to develop a universal immunotherapy against HIV virus. Inclusion of HIV-2 sequences could alter the degree of protein sequence conservation found by others who limited their analysis to HIV-1 sequences. Despite the current high conservation shared amongst HIV-2 strains and the lesser pathogenicity compared to HIV-1 strains, HIV-2 virus should be carefully monitored after reports of faster evolution of Env proteins in patients infected with HIV-2 than those infected with HIV-1 [22]. These results suggested that the HIV-2 virus has the potential to become more pathogenic in the future due to mutations in its genetic material.

The variability of the HIV proteome has been extensively studied by many groups [23-25]. These studies have regularly identified Gag, Pol and Tat as the most conserved HIV proteins, and these sequences together with envelope glycoprotein sequences (Env) are the most commonly incorporated in candidate vaccines [13-19]. In our analysis, we defined highly conserved domains as regions between 20–50 aa where every single consecutive aa was present in ≥70% of the HIV isolate population analysed. Others have used different parameters to determine conservation, such as aminoacid entropy [26].

Domains containing a high number of *in silico* predicted T cell epitopes were defined as those which, according to our in-house algorithm, contained at least 5 CD8+ T cell epitopes for HLA alleles A*02, A*24, B*27 and B*35. These are the most frequently reported HLA Class I alleles worldwide [27,28]. We have used this same approach to select the targets for a novel universal Influenza vaccine (FLU-v), which has successfully completed Phase I / II clinical trials [29,30]. Again, these results are different from those reported in other studies, but those studies also used a different range of HLAs (e.g. as B*07 and A*02, A*11, A*30, etc.) and usually sought to identify naturally occurring immune responses [31].

**Figure 1:**
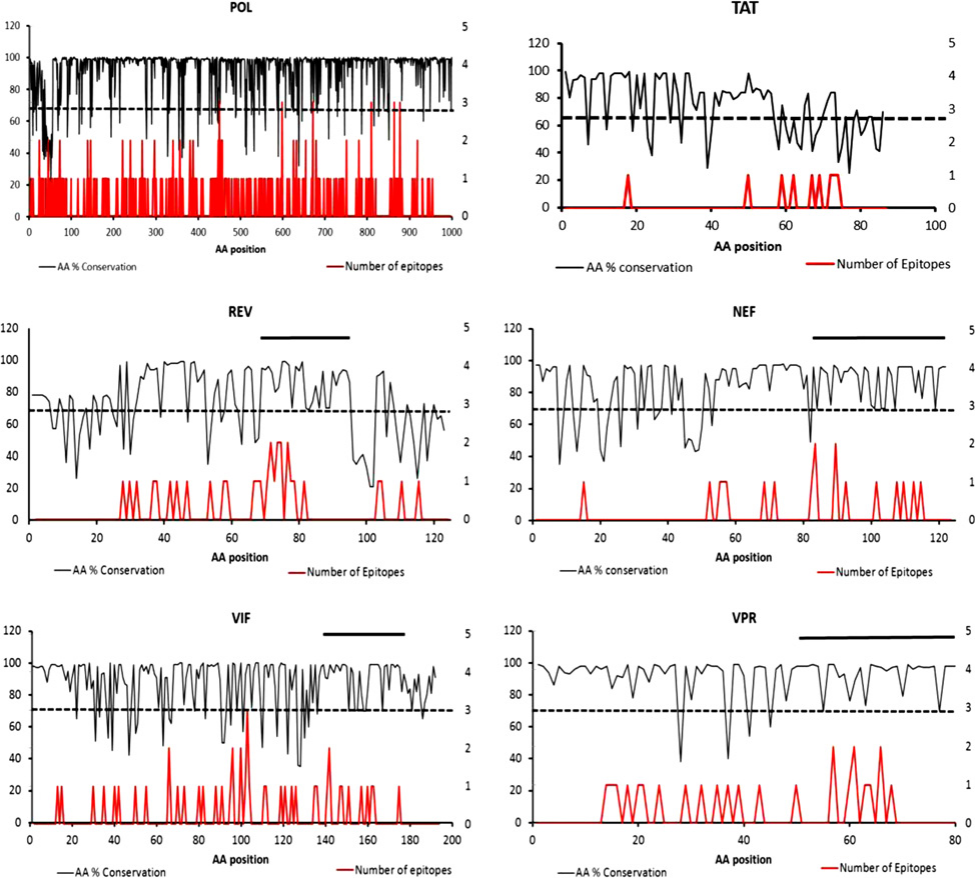
**Sequence variability and CD8+ CTL epitope density in HIV proteins.** Sequence variability and CD8+ CTL epitope density in HIV proteins. Multiple sequence analysis of all HIV-1 and −2 isolates sequences available at the National Centre for Biotechnology Information (NCBI) Taxonomy database (January 2006) were carried out using CLUSTALW. The upper black line represents the % aminoacid conservation along the sequence of selected HIV proteins. The dotted black line represents the minimum conservation threshold (70%) required for the aminoacids within any given region to be considered as conserved. The location of potential CD8+ CTL epitopes was identified *in silico* using a proprietary algorithm. The lower red line represents the number of identified 9-mer epitopes, in the context of HLA A-02, *A-24, *B-27 and *B35, whose first aminoacid falls at the indicated position within the analysed protein.

The results of some of our analysis combining aminoacid conservation and prediction of T-cell epitopes are illustrated in Figure 1. Consistent with the existing literature, proteins such as Pol and Tat (Figure 1) were found to be highly conserved overall and predicted to be most immunogenic. Nonetheless, their conserved regions were rejected due to length (i.e. <20-50 aa), lack of reactive epitopes (i.e. <5), potential for cross-reactivity with other known human or rodent protein sequences (high similarity in at least 7 consecutive aminoacids), and/or unfeasible large-scale synthesis by Fmoc chemistry. Interestingly, we did not encounter these problems with the Vif, Vpr, Rev and Nef proteins (Figure 1). These proteins, despite in several cases having lower levels of conservation, all contained at least one highly conserved region of between 20–50 aa, displaying at least 5 CD8+ T cell epitopes, sharing no similarity with either human or rodent sequences and being amenable to easy large-scale manufacture by F-moc chemistry.

We must point out that our proprietary algorithm identifies and ranks T-cell epitopes within a given protein sequence based on the analysis of the structural affinity of a peptide for a given HLA allele and the potential reactivity of this complex to T cells. Many of the highest ranked epitopes predicted by our algorithm have already been described experimentally and were found within proteins such as Tat and Pol. However, many of these epitopes fell within regions of high variability, probably reflecting immune selective pressure. In contrast, many of the T-cell epitopes predicted in Vif, Vpr, Rev and Nef were ranked as sub-dominant by our algorithm, but they were found within regions of reduced sequence variability. Typically, the bulk of the CD8+ T-cell response is directed to a limited number of immunodominant epitopes [32], and it is probably due to the low frequency of CD8+ effectors to most of the epitopes in Vif, Vpr, Rev and Nef in infected humans that not all these epitopes have been described experimentally. Nonetheless, immunodominance is not a pre-requisite for vaccine efficacy, and subdominant epitopes have already been proposed as candidate targets for therapeutic vaccination against HIV [33,34] and shown to constitute effective vaccines for the control of respiratory viral infections [35,36].

Based on these results, the four identified regions within the regulatory protein Rev and accessory proteins Vif, Nef and Vpr were selected as target sequences for our final vaccine formulation, HIV-v (Table 1). These four proteins are involved in viral replication, immune downregulation and virion assembly [37-41] and other conserved epitopes have been previously described in Vif [42,43], Nef [44-46], Vpr [47] and Rev [48].

**Table 1:** Conserved HIV protein regions containing multiple epitopes

**Name**	**Consensus sequence**	**Location**^**a**^	**Length**
VPR	GDTWAGVEAIIRILQQLLFIHFRIGCQHSR	51 to 80	30
VIF	KVGSLQYLALTALITPKKIKPPLPSVKKLTEDRWNKPQKT	142 to 181	40
REV	EPVPLQLPPLERLTLDCSEDCGTSGTQ	69 to 95	27
NEF	YKGALDLSHFLKEKGGLEGLIYSQKRQDILDLWVYHTQGYFPD	81 to 123	43
M1A*	DLEALMEWLKTRPILSPLTKGILGFVFTLTVP	36 to 67	32
M1B*	LLYCLMVMYLNPGNYSMQVKLGTLCALCEKQASHS	124 to 158	35
NPA*	DLIFLARSALILRGSVAHKSC	255 to 275	21
NPB*	PGIADIEDLTLLARSMVVVRP	306 to 326	21
PB1*	LLIDGTASLSPGMMMGMFNMLSTVLGVSILNLGQ	395 to 428	34
M2*	IIGILHLILWILDRLFFKCIYRLF	32 to 55	24

^a^Amino acid location for each sequence is referenced to the consensus sequence defined for that protein. The length of the polypeptide is expressed as number of aminoacids. Marked with (*) are the aminoacid sequences component of NRP-v.

### Immunogenicity of selected polypeptides

To establish whether HIV-v could induce an antigen specific cellular response within the context of human MHC, C57BLK/6 mice transgenic for Class I HLA-A*0201 were immunised with either HIV-v or a non-relevant polypeptide formulation (NRP-v). These mice express HLA-A2.1 in spleen, bone marrow and thymus and have been used as a model for the identification of HLA-A2.1 restricted CD8+ CTL epitopes in other virus [49]. HIV-v was prepared as an emulsion prior injection with adjuvant Montanide ISA-51. A preliminary study concluded that adjuvant Montanide ISA-51 was required to maximise the immunogenicity of the HIV-v preparation (data not shown). Montanide ISA-51 is composed of a light mineral oil and a surfactant system designed to make a water-in-oil emulsion. It has been demonstrated to be a very efficient adjuvant, activating the cellular and the humoral immune response [50,51]. Comparative studies with calcium phosphate gel and aluminium-based adjuvants suggest that Montanide ISA-51 is amongst the safest and most effective adjuvants for synthetic peptide vaccine formulations [8]. Montanide ISA-51 is part of the growing number of adjuvant formulations being developed for human use. In HIV these include lipopeptide formulations of Nef, Gag and Env derived peptides or full proteins which have been shown to elicit sustained T cell responses in clinical trials [52,53].

**Figure 2:**
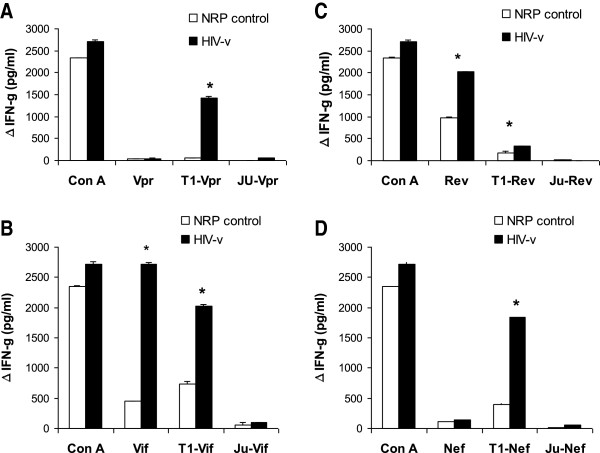
**IFN-γ secretion by splenocytes exposed to soluble and transfected HIV-v polypeptides.** Splenocytes of HLA-A*0201 transgenic mice immunised with HIV-v or NRP-v were exposed *in vitro* to Vpr (**A**), Vif (**B**), Rev (**C**) and Nef (**D**) polypeptides as well as polypeptides transfected in syngeneic T1 (T1-Vpr, T1-Vif, T1-Rev and T1-Nef) and allogeneic JURKAT (Ju-Vpr, Ju-Vif, Ju-Rev and Ju-Nef) cells. IFN-γ production is represented as the net increment in IFN-γ production (pg/ml) over the response to the negative control antigens Lysozyme or non-transfected cells (25 ±10 pg/ml for soluble Lysozyme, 316 ±43 pg/ml for T1 cells, and 19 ±6 pg/ml for JURKAT cells, average ± SEM). Con A was used as a standard positive control to confirm assay validity. A positive response, indicated by an asterisk (*), was defined as an increment of at least 30% over the control group’s response with a statistical significance of p < 0.05. Splenocytes from each individual were assayed separately in quadruplicate wells. This graph is a representative of three independent experiments.

In our studies, splenocytes from HIV-v immunised animals secreted higher levels of IFN-γ (p <0.05) than those from NRP-v immunised animals when cultured with soluble Vif (2721.3 ± 22.7 vs 446.7 ± 10.6, pg/ml, mean ± S.E.M) and Rev (2027.5 ± 8.0 vs 976.3 ± 10.5) polypeptides (Figure 2). Very little IFN-γ secretion was observed in response to soluble Vpr and Nef polypeptides in splenocytes from either HIV-v or NRP-v immunised mice. This may reflect their reduced solubility in cell culture media, and hence bioavailability, and/or the absence of strong murine T cell epitopes in their sequences.

*In vitro* stimulation with soluble antigen is regularly used to determine overall antigen-specific cellular immune responses. However, because our HLA-A*0201 transgenic mice strain expresses simultaneously Class I murine MHC and HLA molecules, the assay is unable to discriminate between T cells reacting to epitopes complexed to murine MHC and those reacting to epitopes complexed to HLA. Moreover, because murine Class I MHC molecules are more abundant on the cell membrane of these mice than Class I HLA molecules, epitopes derived from soluble antigen captured and processed by APCs in the splenocyte suspension have a greater likelihood of being complexed with the murine molecules [49]. To address this problem and to allow us to identify HIV-v specific Class I HLA restricted T cell responses, single polypeptides were also transfected into syngeneic (HLA-A*0201 bearing) T1 or allogeneic (HLA-A*0201 non-bearing) JURKAT human cells. Co-culture of splenocytes from HIV-v or NRP-v immunised animals with allogeneic Jurkat cells transfected with HIV-v polypeptides did not induce significant levels of IFN−γ secretion. However when the same splenocytes were co-cultured with syngeneic T1 cells transfected with HIV-v polypeptides, a significant increase (p <0.05) in IFN−γ secretion (pg/ml, mean ± S.E.M) was detected in the HIV-v vaccinated animals (HIV-v vs NRP-v immunised mice: Vpr: 1413.5 ± 43.4 vs 52.8 ± 7.3; Vif: 2027.8 ± 22.2 vs 737.4 ± 39.7; Rev: 329.3 ± 10.5 vs 172.2 ± 40.2; Nef: 1843.8 ± 15.5 vs 391.9 ± 25.7, respectively) (Figure 2).

HLA-A*0201 transgenic mice do not bear any other HLA and there is no evidence that their CD8+ T cells would recognize any HIV-v-derived epitopes in the context of other HLAs that they have never encountered [54]. Therefore, an increased IFN-γ production by transgenic splenocytes from HIV-v vaccinated animals when co-cultured with human syngeneic cells transfected with polypeptide, but not with transfected allogeneic cells, is interpreted as a response mediated by CD8+ T cells recognising HIV-v epitopes via HLA-A*0201.

No IL-4 responses were detected against any of the polypeptides, either soluble or transfected (data not shown). Since IL-4 antagonises IFN−γ, the lack of an IL-4 response is again consistent with HIV-v inducing a Th1-like response.

### Cellular response to HIV-infected human cell lines

Having demonstrated that HIV-v immunised splenocytes recognise HIV-v derived epitopes in an HLA Class I restricted manner, we wished to determine whether they were also capable of recognising virus derived epitopes that are naturally processed and complexed to Class I HLA molecules in HIV infected human cell lines. For this purpose, HIV-v and NRP-v splenocyte suspensions were co-cultured *in vitro* with syngeneic (T1) and allogeneic (H9) human cell lines, either alone or infected with HIV-1 IIIb, and tested by flow cytometry for IFN−γ production by CD3 + CD8+ T cells.

**Figure 3:**
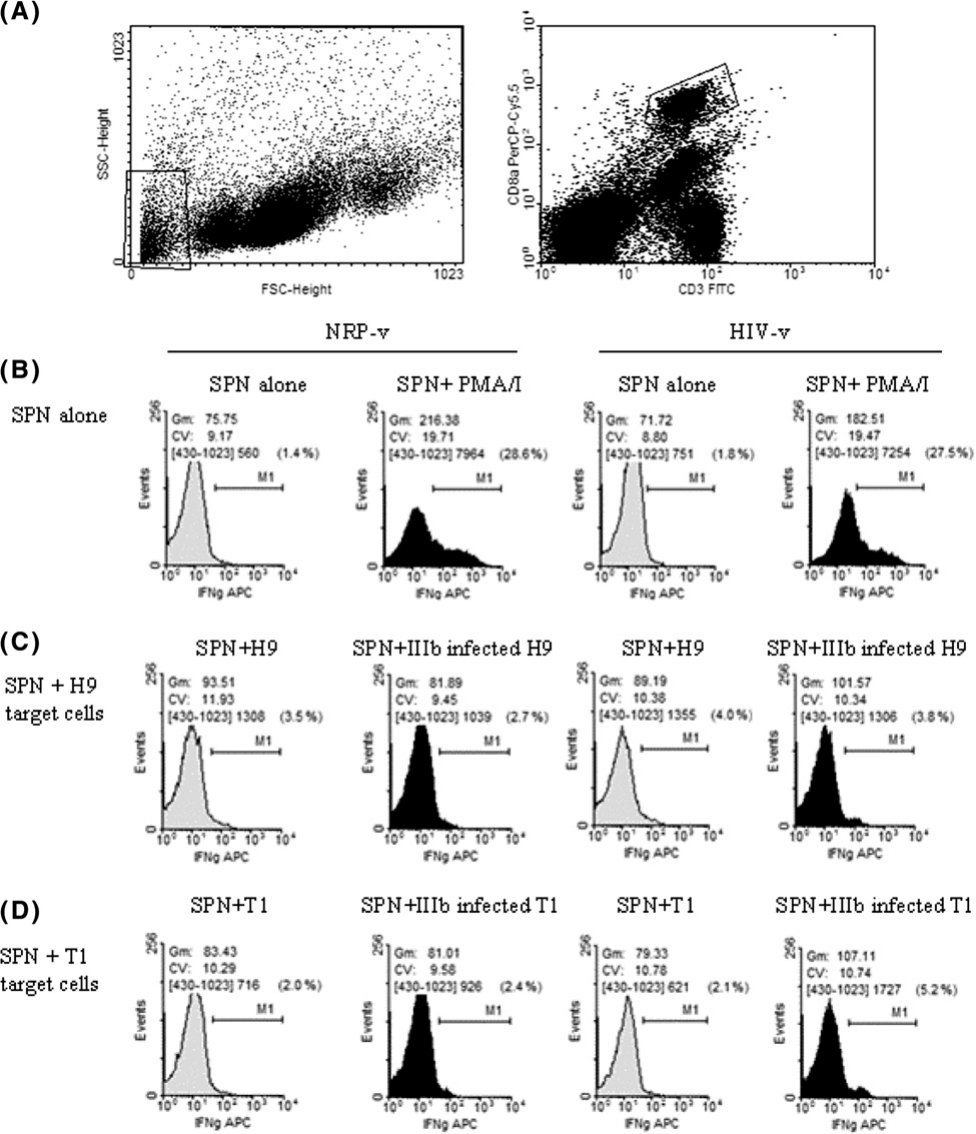
**IFN-γ production by CD3 + CD8+ cells in response to co-culture with HIV-infected human cells.** IFN-γ responses of primary splenocyte cultures from HLA-A*0201 transgenic mice immunised with either HIV-v or NRP-v following co-culture with human syngeneic (T1) or allogeneic (H9) cells alone or infected with the HIV-1 IIIB strain were measured by flow cytometry. Panel **A** indicates the gating strategy applied to the analysis. Non viable cells and target cells were gated out prior measuring IFN-γ in CD3 + CD8+ cells. Panels **B** to **D** show the number of IFN-γ producing cells (events Y axis) vs the intensity of IFN-γ produced (X axis). The marker (M1) corresponds to the range of signal considered as a positive response. The response’s Geometric Mean (GM) and coefficient of variation (CV) as well as the % of events within M1 are also provided. Panel **B** corresponds to splenocytes (SPN) from NRP-v and HIV-v immunized groups untreated or treated with PMA + Ionomycin (PMA/I). Panel **C** corresponds to splenocytes co-cultured with allogeneic H9 alone or infected with IIIb HIV-1. Panel **D** corresponds to splenocytes co-cultured with syngeneic T1 cells alone or infected with IIIb HIV-1.

In the positive control, stimulation of HIV-v and NRP-v splenocytes with PMA-Ionomycin (PMA/I) increased the percentage of IFN-γ producing CD3 + CD8+ T cells by over 15 fold and the mean IFN-γ produced per cell by over 200% in both groups (Figure 3). Co-culture of HIV-infected or non-infected allogeneic human H9 cells with splenocytes from HIV-v and NRP-v vaccinated mice did not differ in either the number of IFN-γ producing CD3+ CD8+ cells or in the mean IFN-γ produced per cell (Figure 3). Similarly, no differences were observed in splenocytes from the NRP-v group co-cultured with either healthy or HIV infected syngeneic T1 cells. In contrast, splenocytes from the HIV-v immunised group co-cultured with HIV-infected syngeneic T1 cells experienced a 2-fold increase in the number of CD3+ CD8+ IFN-γ producing cells and a 35% increment in the mean IFN-γ produced per cell, compared to co-culture with non-infected cells (Figure 3). The response generated by HIV-v vaccination at this stage is modest, but supports the conclusion that HIV-v immunisation can induce HLA-A*0201 restricted CD3 + CD8+ T cells that specifically recognise an HIV infected human cell line. Improvements in this response might be achieved through optimisation of the dose and the schedule of immunisation.

The slightly higher level of the CD3 + CD8+ background response observed in both the HIV-v and NRP-v groups against the non-infected H9 cells compared to non-infected T1 cells (non-infected H9 vs T1; HIV-v: 4.0% v 2.1%; NRP-v: 3.5% v 2.0%) is not biologically relevant to the virus-specific response elicited by HIV-v vaccination. This difference may be explained from the underlying non-antigen specific stimulatory response triggered by the allogeneic nature of the H9 cells and is consistent with previous observations reported in the literature [55].

### Antibody response to HIV-v

Antibodies play multiple roles in anti-viral immunity including virus neutralisation [56], immune complex phagocytosis [57], complement activation [56,58] and antibody-dependent cellular cytotoxity (ADCC) [56,59,60]. Antibodies are distributed in serum and the extravascular space of mucosal tissues [61,62], one of HIV’s primary routes of entry and hence can play a key role in controlling infection and disease. However generation of anti-HIV antibody responses is delayed until virus latency is established. Early destruction of B cell generative microenvironment may be responsible for this delayed antibody response [63].

**Figure 4:**
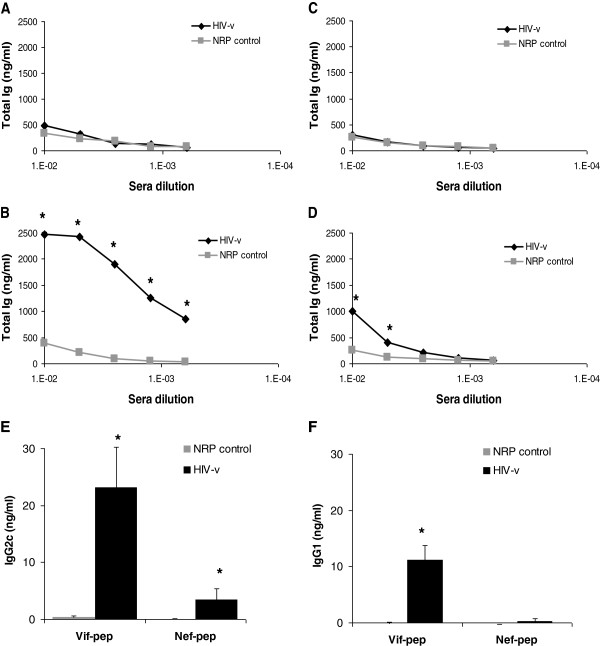
**Total Ig and IgG isotype responses to the HIV-v polypeptide components.** Responses were measured by ELISA in sera from HIV-v or NRP-v immunised HLA-A*0201 transgenic mice. Total Ig responses to the Vpr (**A**), Vif (**B**), Rev (**C**) and Nef (**D**) peptides are represented as average ng/ml (± SEM) versus sera dilution. IgG2c (**E**) and IgG1 (**F**) responses to Vif and Nef peptides were quantified in a 1:200 serum dilution. A positive response, indicated by an asterisk (*) was defined as an increment of at least 100% over the background response with a statistical significance of p < 0.05.

Our *in silico* algorithm does not assess, and the HIV-v polypeptides were not selected based on B-cell immunogenicity. Nonetheless, HIV-v vaccination elicited an IgG response specific to Vif and Nef polypeptides (Figure 4B and 4D). Interestingly, the response to the Vif polypeptide was higher (still detectable at a 1/1600 serum dilution, p < 0.05) than that to the Nef polypeptide. No response against the Vpr or Rev polypeptides was observed (Figure 4A and 4C). Further analysis of the Vif and Nef responses determined that the IgG2c component is dominant over the IgG1 (Figure 4E and 4F).

HIV patients generally produce antibodies against Vpr and Rev [64,65] and the lack of response in our study may reflect the absence of B cell epitopes in the selected Vpr and Rev regions. The isotype of the IgG response was biased towards the IgG2c subclass, which is functionally equivalent to IgG2a in the genetic background of the transgenic mice used [66]. This subclass is associated with IFN-γ production [67] which is consistent with our assertion that HIV-v immunisation triggers a Th1 immune response.

### HIV-v specific antibodies induce lysis of HIV-infected T1 cells through complement activation

The trend in HIV vaccine development has focused for many years on the development of an antibody response characterised by the generation of neutralising antibodies (NAbs) directed to viral capsid components. These NAbs naturally appear months after infection and are not able to neutralise viral strains divergent from the infecting strain [68]. Recent efforts to develop more broadly neutralising antibodies (bNAbs) have proven difficult [69]. A different strategy is to develop a non-neutralising antibody responses that are directed not to virion components (e.g. capsid proteins), but to viral proteins that are associated to the cellular membrane of infected cells. These antibodies would then be able to trigger either ADCC via the Fc receptors on NK and Macrophages of effector cells or complement activation. In either case the final outcome would be the destruction of the HIV-infected cells [70-72].

**Figure 5:**
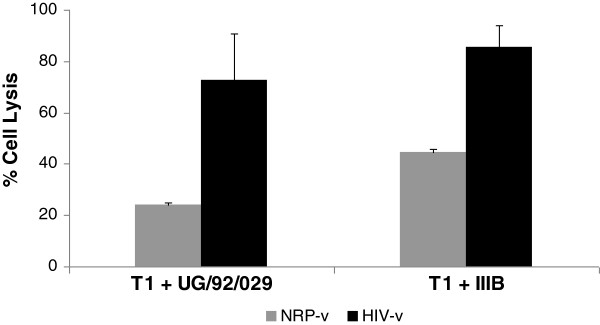
**Complement mediated cell lysis of HIV-1 infected human cells measured as LDH release.** Heat inactivated sera from HIV-v and NRP-v immunised mice were diluted 1/100 in PBS, combined with rabbit complement and added to cultures of T1 cells infected with HIV-1 field isolate UG-29-029 (clade A) or laboratory strain IIIB. The percentage of cell lysis was defined between the level of LDH release of non-infected T1 cells in the presence of complement without sera (0%) and the LDH released after lysing the same cells with Triton X-100 (100%). A positive response, indicated by an asterisk (*), was defined as an increment of lysis of at least 50% over that of HIV-1 infected cells in the presence of control (NRP-v) sera and with a statistical significance of p < 0.05. Represented are the averages of percentage cell lysis ± SEM.

HIV-v targets primarily non-capsid proteins and thus the antibody response it elicits is not expected to have neutralisation potential. Nonetheless, we wished to establish whether the IgG response generated against HIV-v could mediate a significant anti-viral effector mechanism. For that purpose, NRP-v and HIV-v immunised sera were tested for their ability to activate complement and lyse human syngeneic T1 cells infected with HIV strain IIIB or clade A field isolate UG/92/029. Figure 5 shows that sera from HIV-v immunised animals induced a significantly higher level of lysis of HIV infected T1 cells than sera from NRP-v immunised animals (72.8% ± 17.8 vs 23.9% ± 1.1 for UG/92/029 infected cells, and 85.7% ± 8.2 vs 44.8% ± 1.0 for IIIB infected cells).

The different levels of cell lysis induced by the NRP-v sera against cells infected with the two test strains of HIV-1 do not detract from the clear anti-viral effector function of the HIV-v specific sera, but they do reflect two important features of the assay. Firstly, the baseline lysis (0%) for both the IIIB and the UG/92/029 infected T1 cells is defined by non-infected T1 cells in the presence of complement. Viral antigens on the surface of infected cells are known to spontaneously activate the alternative complement pathway, which is antibody independent [73]. As a result, the observed increase over baseline in the lysis caused by NRP-v is probably due to a nonspecific release of LDH enzyme during the assay. Secondly, in preparation for this assay, T1 cells were infected with the different viral strains and only used as targets when intracellular HIV p24 expression was maximal. For strain IIIB that corresponded to 2 days post-infection (42% cellular viability) whilst for strain UG/92/029 it was 10 days post-infection (61.2% cellular viability). As the assay measures release on the medium of a strict intracellular enzyme (LDH), which is also known to leak through damaged membranes, the higher non-specific response (i.e. against NRP-v) observed against the IIIb infected T1 cells (44.8%) is consistent and entirely correlates with its reduced cell viability (42%) at the point of maximum HIV p24 expression.

## Conclusion

In summary, immunisation with HIV-v, an equimolar formulation of four synthetic polypeptides covering conserved immunogenic regions in Vif, Vpr, Nef and Rev in Montanide ISA-51, induces specific CD8+ T cell and IgG responses that are able to recognise and kill a human cell line infected with either laboratory adapted or primary isolate strains of the HIV-1 virus. Based on our results, we believe HIV-v constitutes a promising HIV vaccine and/or immunotherapeutic candidate. Moreover, the cytotoxic effect observed for the antibodies produced against Nef and Vif peptides highlights their potential value for the development of passive immunotherapeutic agents (e.g. MAb polytherapy) to treat HIV infection.

## Methods

### Animals, cell lines and virus

Transgenic homozygous C57BL/6-TgN(HLA-A2.1)1Enge mice, male: female ratio 1:1, aged 7–10 weeks, weighing between 22-23 g (males) and 18-21 g (females) were used. Transgene carrier status and expression were monitored and confirmed every 6 months by qPCR and RT-PCR, respectively at Charles River, UK.

All cell lines were obtained from ATCC. T1 (HLA-A*0201-bearing) and H9 (non-HLA-A*0201-bearing) human cell lines were maintained in IMDM (Sigma) whilst JURKAT (non- HLA-A*0201-bearing) and splenocytes were kept in RPMI-1640 (Sigma). Media was supplemented with 50 IU/50 μg/ml of penicillin/streptomycin (Sigma) and 10% FCS (20% for H9 cells)(Sigma).

HIV-1 strain IIIB or field isolate UG/92/029 (Clade A) (NIBSC) were used to naturally infect T1 and H9 cells. Infection was monitored with intracellular p24 Gag level. Optimum infection was achieved after 2 days in IIIB infected cells (42% cellular viability) vs 10 days in UG/92/029 infected cells (61.2% cellular viability).

### Ethics

All animal experimentation was carried out by third parties. Southern Research Institute (United States) carried out the work according to IACUC protocols (US) under ethical approval granted to projects 12761.01, 12595.01 and 12595.02.

The experiments carried out at Keele University (United Kingdom) and Harlan UK were done under project licenses PPL 40/2411 and PPL 60/3418, respectively, following the European Directive EC 86/609 and her majesty’s Home Office regulations under the Animals (Scientific Procedures) Act 1986.

### Identification of conserved immunoreactive regions

Conserved regions in HIV were identified by analysing with CLUSTALW [74,75] all HIV-1 and −2 isolates sequences available at the National Centre for Biotechnology Information (NCBI) Taxonomy database (January 2006) [76]. The aim of the analysis was to determine the existence and location of short highly conserved domains within the HIV proteome and not the overall degree of conservation of individual proteins. Regions of 20 to 50 aa in the consensus sequences where every consecutive amino acid was present in ≥70% of the isolate population were considered conserved. The identified consensus sequences were analysed for the presence of reactive T cell epitopes for mouse MHC H-2kb and HLA *A-02, *A-24, *B-24,*B-27 and *B35 alleles using a proprietary algorithm (PepTcell Ltd). The algorithm identifies and categorises T cell epitopes within a protein based on analysis of the structural affinity of a peptide for a given MHC/HLA allele and the reactivity of this complex to T cells. The final selection of conserved polyepitope T-cell reactive fragments in the HIV population was based on four criteria: 1) length between 20–50 aa long, 2) containing at least five reactive epitopes, 3) sharing no similarity to other murine/human protein sequences, and 4) feasibility of F-moc synthesis. The final successful candidates were synthesised by Fmoc chemistry at BACHEM (Switzerland).

### Immunisations

HIV-v is an equimolar mix of four multi-epitope synthetic polypeptides. NRP-v is an equimolar mix of multi-epitope non-HIV derived polypeptides. On Day 1 mice were immunised subcutaneously at the base of the tail (200 μl) with HIV-v or NRP-v (10 nmol of each peptide, optimal for T cell response, or 5 nmol of each peptide, optimal for antibody response) in PBS emulsified 1:1 with adjuvant Montanide ISA-51 (Seppic). A total of 12 animals (6 males and 6 females) per group were immunised. All animals received a booster immunisation (same doses) on day 15 and were culled on day 21 when spleens and sera were collected. All experimental work was carried out in accordance with the Scientific Procedures Act (1986) and IACUC regulations.

### Cytokine ELISA from supernatants of splenocyte cultures

Splenocyte suspensions from each single animal were prepared as previously described [29], seeded at 4 × 10^5^ cells/well in 96-well plates (BD) and cultured in quadruplicates for 96 h with either soluble treatments (i.e. media, 5 ug/ml of NRP-v, 5 ug/ml of each individual HIV-v polypeptide, 5 ug/ml Concanavalin A (Sigma), 1ug/ml Lysozyme (Sigma)), or 4×10^4^ cells (T1 or Jurkat) either alone or transfected with each individual HIV-v polypeptide. Transfections were performed using Lipofectin (Invitrogen) following manufacturer’s instructions. IFN-γ and IL-4 responses were determined by ELISA analysis of the supernatants according to the manufacturer’s instructions (OptEIA kits, BD). This experiment was performed three times.

### Intracellular cytokine staining

Splenocyte suspensions were seeded at 4×10^6^ cells/ml and challenged with either 10 ng/ml PMA + 1 μg/ml Ionomycin (Sigma) or 4×10^5^ cells/ml of Mitomycin C-inactivated T1 (syngeneic) or H9 (allogeneic) cells, either alone or infected with HIV-1 IIIB. Following 15 h incubation at 37°C, GolgiPlug (BD) was added and plates were incubated for a further 4 h. Supernatants were removed, a FcR block performed and the cells washed and stained with anti-mouse CD3 FITC-conjugated (clone 145-2C11) and anti-mouse CD8 PerCP-Cy5.5-conjugated (clone 53–6.7) antibodies (BD) for 15 min. After washing, cells were fixed for 20 min (Cytofix/Cytoperm, BD) and washed again. After permeabilisation for 20 min with Perm wash buffer (BD), cells were washed and anti-mouse IFN-γ APC-conjugated (clone XMG1.2, BD) antibody was added. After 20 min incubation, cells were washed and resuspended in Cytofix buffer (BD). After overnight storage at 4°C cells were analysed with a FACScalibur flow cytometer (BD). One million events were acquired per reaction and data was processed using WinMDI 2.9 software. Non-viable cells were gated out in a FSC/SSC. Viable cells were plotted in a CD3vsCD8 dot plot to separate the CD3 + CD8+ splenocytes from the HIV-infected target cells. Intracellular IFN-γ was then measured in the CD3 + CD8+ splenocyte population.

### Antibody ELISA

ELISA 96-well plates were coated overnight at +4°C with 2 μM of single HIV-v polypeptides in PBS (Sigma). Plates were washed with PBS + 0.05% Tween 20 (Sigma) (PBS-T) and blocked for 1h with 1% BSA Fraction V (Sigma) in PBS. After washing with PBS-T, test sera samples were added. Following 2 h incubation, plates were washed with PBS-T and either HRP-conjugated goat anti-mouse-Ig (Sigma), HRP-goat anti-mouse IgG1 (AbD Serotec) or HRP-rat anti-mouse IgG2a/c (BD Biosciences) was added. After 1h incubation, plates were washed with PBS-T and TMB substrate (Sigma) was added. The reaction was stopped with 0.5M H_2_SO_4_ and absorbance was read at 450 nm. Antibody concentrations were quantified against purified total Ig (Sigma), IgG2c (BD Biosciences) and IgG1 (AbD Serotec) standards. Sera samples from each individual were tested separately in triplicates at various dilutions (1:100, 1:200, 1:400, 1:800 and 1:1600).

### Antibody-activation of complement

HIV-1 IIIb or UG/92/029 (Clade A) infected T1 cells were seeded at 6×10^3^ cells/well in flat bottom 96-well plates together with heat inactivated test sera (diluted 1/100 in PBS) and baby rabbit complement (AbD Serotec). After 2 h incubation, cell lysis (Experimental Release) was measured using the LDH based CytoTox 96®Non-Radioactive Cytotoxicity Assay according to manufacturer’s instructions (Promega). The dynamic range of the assay was determined using as minimum LDH release (MIN) non-infected T1 cells incubated with baby rabbit complement in PBS without sera and as maximum LDH release (MAX) cells lysed with Triton X-100.

The percentage of Specific Immune Lysis (% SIL) was calculated using the following formula:

$$\mathrm{\%SIL}=100*\frac{\mathrm{ExperimentalRelease}-\mathrm{MINRelease}}{\mathrm{MaxRelease}-\mathrm{MINRelease}}$$

### Statistical analysis

Statistically significant increases in the immune responses to antigens between the HIV-v and NRP-vaccinated animals were established by non-parametric Mann–Whitney analysis. Differences were considered statistically significant if the p value was <0.05.

## Abbreviations

HIV: Human Immunodeficiency Virus; HIV-v: HIV vaccine: NRP-v, Non Related polypeptide vaccine; aa: aminoacid; IFN-γ: Interferon gamma; IL-4: Interleukin-4; BSA: Bovine Serum Albumin; HRP: Horse Radish Peroxidase; Ig: Immunoglobulin; HLA: Human Leukocyte Antigen; MHC: Major Histocompatibility Complex; CTL: Cytotoxic T lymphocyte; PMA: Phorbol Myristate Acetate

## Competing interests

This study was fully sponsored by SEEK (Peptcell Ltd), a privately owned biopharmaceutical company. Wilson Caparrós-Wanderley and Gregory Stoloff hold shares in the company. A patent covering the peptide sequences and the compositions was published under number WO2007104932.

## Authors’ contributions

WC-W and GAS identified the predicted immunoreactive regions of HIV-v. OP and WC-W contributed to the vaccine preparation, design, acquisition and analysis of the polypeptide immunogenicity data. OP perfomed the isotyping of the immune response to HIV-v and optimised complement assays. OP, WC-W and GAS drafted and reviewed the manuscript. All authors read and approved the final manuscript.
